# Cloning, Expression Analysis and Functional Characterization of Squalene Synthase (SQS) from *Tripterygium wilfordii*

**DOI:** 10.3390/molecules23020269

**Published:** 2018-01-29

**Authors:** Bin Zhang, Yan Liu, Mengmeng Chen, Juntao Feng, Zhiqing Ma, Xing Zhang, Chuanshu Zhu

**Affiliations:** 1 Research & Development Center of Biorational Pesticides, Northwest A & F University, Yangling 712100, China; zhangbin1990@nwsuaf.edu.cn (B.Z.); liuyan0848@163.com (Y.L.); cym135266@163.com (M.C.); fengjt67@hotmail.com (J.F.); zhiqingma@nwsuaf.edu.cn (Z.M.); 2 Biopesticide Technology and Engineering Center of Shaanxi Province, Yangling 712100, China

**Keywords:** *Tripterygium wilfordii*, squalene synthase, celastrol, prokaryotic expression, functional characterization

## Abstract

Celastrol is an active triterpenoid compound derived from *Tripterygium wilfordii* which is well-known as a traditional Chinese medicinal plant. Squalene synthase has a vital role in condensing two molecules of farnesyl diphosphate to form squalene, a key precursor of triterpenoid biosynthesis. In the present study, *T. wilfordii* squalene synthase (TwSQS) was cloned followed by prokaryotic expression and functional verification. The open reading frame cDNA of *TwSQS* was 1242 bp encoding 413 amino acids. Bioinformatic and phylogenetic analysis showed that TwSQS had high homology with other plant SQSs. To obtain soluble protein, the truncated TwSQS without the last 28 amino acids of the carboxy terminus was inductively expressed in *Escherichia coli* *Transetta* (DE3). The purified protein was detected by SDS-PAGE and Western blot analysis. Squalene was detected in the product of in vitro reactions by gas chromatograph-mass spectrometry, which meant that TwSQS did have catalytic activity. Organ-specific and inducible expression levels of *TwSQS* were detected by quantitative real-time PCR. The results indicated that *TwSQS* was highly expressed in roots, followed by the stems and leaves, and was significantly up-regulated upon MeJA treatment. The identification of TwSQS is important for further studies of celastrol biosynthesis in *T. wilfordii*.

## 1. Introduction

*Tripterygium wilfordii* (Celastraceae) is an important Chinese herbal medicine used for the treatment o f inflammation [1]. Active ingredients of *T. wilfordii* include sesquiterpenoid alkaloids, diterpenoid, and quinone-methide triterpenoid (QMTs) with varied pharmaceutical activities [2]. Many QMTs have already been isolated from Celastraceae, such as celastrol, maitenin, 22-hydroxytingenone and pristimerin [3]. They have similar chemical structures and biological activities. Celastrol is the first active compound isolated from the root bark of *T. wilfordii* and also is one of the major active ingredients of *T. wilfordii* [4]. As a representative compound of QMTs, Celastrol has been demonstrated to have promising activities including antioxidant [5], anti-inflammatory [6], immunomodulation [7], and anticancer activities [8]. Celastrol has shown promise as an anti-inflammatory compound for arthritis, lupus, amyotrophic lateral sclerosis, and Alzheimer’s disease [6]. Recent studies have shown that celastrol is a potential therapeutic agent for anti-obesity [9,10]. Celastrol has attracted increased interest recently regarding its therapeutic mechanisms and clinical applications. Traditionally, celastrol is obtained by extraction from the root of *T. wilfordii*. However, *T. wilfordii* is not widely cultivated and the content of celastrol in natural plants is far below demand. The chemical synthesis of this compound is not an economical alternative because of its multistep reactions and low overall yield [11]. Tissue cultivation is a potential choice for the production of natural products, but up to now, only the culture of *T. wilfordii* in a small-scale 10-L bioreactor has been reported [12]. Synthesis on an industrial scale is still far away. Therefore, it is of interest to produce the compound through synthetic biology strategies. However, the development of these strategies requires detailed understanding of the celastrol biosynthetic pathway.

Squalene synthase (SQS, EC 2.5.1.21) is a membrane-associated enzyme that catalyzes the first enzymatic step in sterols and triterpenoid biosynthesis [1]. It converts two molecules of farnesyl diphosphate (FPP) into squalene, which is a precursor to both sterols and triterpenoid [13]. The SQS enzyme is considered to be a pivotal enzyme in the regulation of triterpenoid biosynthesis. Thus, the genes encoding the enzyme have been cloned from several organisms including fungi [14,15], bacteria [16], animals [17], and human beings [18,19]. Botanical squalene synthase enzymes have also been identified in *Arabidopsis thaliana* [20], *Magnolia officinalis* [21], *Salvia miltiorrhiza* Bunge [22], *Panax ginseng* [23], *Panax notoginseng* [24], and *Glycine max* [25]. The squalene synthases identified from *P. ginseng* were able to convert yeast erg9 mutant cells to ergosterol prototrophy in spite of sequence divergence to yeast [21]. Likewise, similar results have been reported in *Glycine max.* The GmSQS1 was able to convert yeast sterol auxotrophy erg9 mutant to sterol prototrophy and overexpression of *GmSQS1* increased end product sterols in *Arabidopsis* seeds [23]. In addition, many SQSs have been investigated subsequently followed by recombinant expression and preliminary enzyme activity. These recombinant SQSs could synthesize squalene from FPP in the presence of NADPH and Mg^2+^ [19,20,22]. Many studies have demonstrated that overexpression of SQS genes could enhance the accumulation of triterpenoid and/or phytosterols compounds in *Withania somnifera* [26], *Panax ginseng* [27], *Eleutherococcus senticosus* [28], *Bupleurum falcatum* [29] and *Solanum chacoense* [30].

According to these studies, we believe that TwSQS may play a pivotal role in celastrol biosynthesis. However, the function of SQS is still unexplored in *T. wilfordii*. Here, we describe the prokaryotic expression, functional characterization and expression analysis of a squalene synthase gene from *T. wilfordii* and establish the foundation to study the celastrol biosynthetic pathway. In addition, the tissue expression of *TwSQS* in *T. wilfordii* natural plants and the expression in *T. wilfordii* hairy root induced by methyl jasmonate (MeJA) were investigated.

## 2. Results

### 2.1. Sequence Analysis of TwSQS

**Figure 1:**
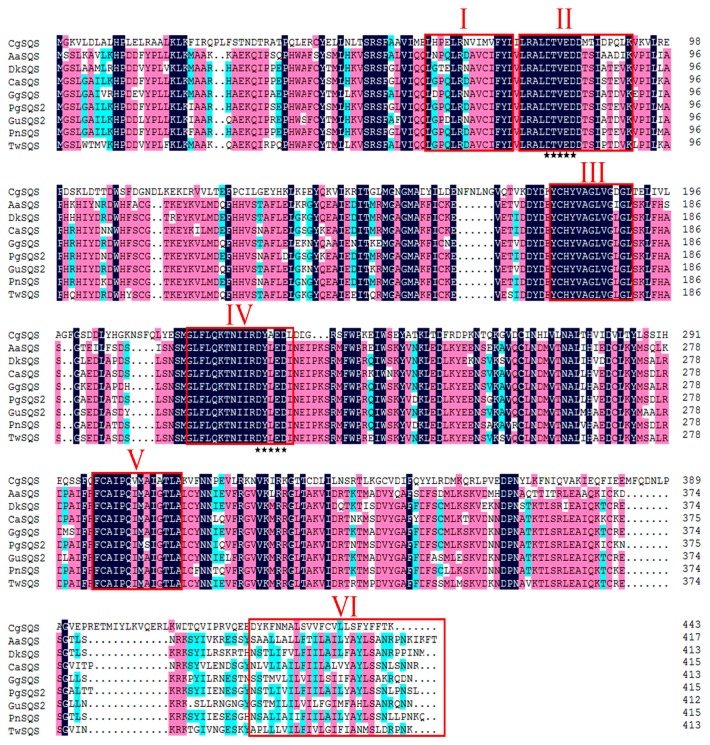
Sequence alignment of TwSQS with squalene synthases from other plants. The accession numbers of the aligned sequences are as follows: AaSQS1, *A. annua*, AAR20329.1; *DkSQS*, *Diospyros kaki*, ACN69082.1; CaSQS1, *C. asiatica*, AAV58897.1; GgSQS, *G. glabra*, BAA13083.1; PgSQS, *P. ginseng*, ACV88718.1; GuSQS2, *G. uralensis*, ADG36719.1, PnSQS, *P. notoginseng,* ABA29019.1, and *Candida glabrata*, BAB12207.1. The six conserved regions (I, II, III, IV, V, and VI) of squalene synthase are marked by “□”, two aspartate-rich domains (DXXXD) that mediate binding of FPP are marked out by “**”.

**Figure 2:**
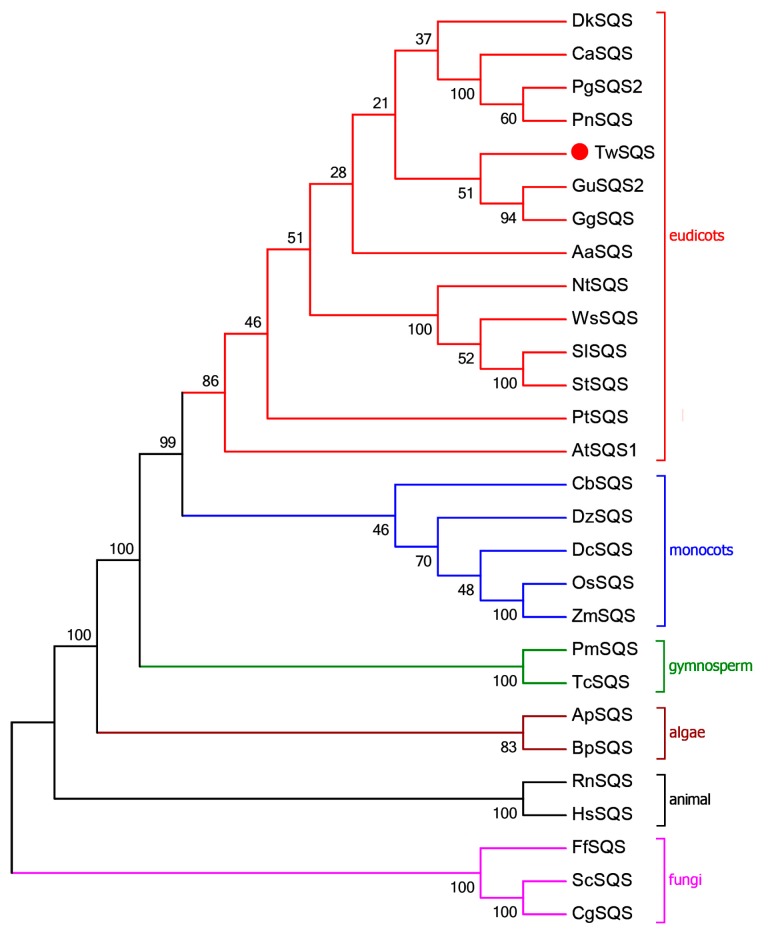
Phylogenetic analysis of TwSQS with other SQSs from diverse organisms constructed by the neighbor-joining method based on 1000 bootstrap replicates. The accession numbers of the aligned sequences are as follows: NtSQS, *Nicotiana tabacum*, AAB08578.1; WsSQS, *W. somnifera*, ADC95435.1; SlSQS, *Solanum lycopersicum*, NP 001234716.2; StSQS, *Solanum tuberosum,* BAA82093.1; PtSQS, *Psammosilene tunicoides,* ABQ96265.1; AtSQS1, *Arabidopsis thaliana,* AEE86403.1; CbSQS, *Chlorophytum borivilianum*, AFN61199.1; DzSQS, *Dioscorea zingiberensis*, AGN32410.1; OsSQS, *Oryza sativa* Japonica Group, BAA22557.1; DcSQS, *Dendrobium catenatum*, AGI56082.1; ZmSQS, *Zea mays*, BAA22558.1; PmSQS, *Pinus massoniana*, AHI96421.1; TcSQS, *Taxus cuspidate*, ABI14439.1; ApSQS, *Auxenochlorella protothecoides*, KFM22694.1; BpSQS, *Bathycoccus prasinos*, CCO17009.1; RnSQS, *Rattus norvegicus*, AAA42179.1; HsSQS, *Homo sapiens*, AAB33404.1; FfSQS, *Fusarium fujikuroi*, ABX64425.2; ScSQS, *Saccharomyces cerevisiae*, AAA34597.1.

The open reading frame (ORF) cDNA of *TwSQS* was amplified by PCR using primers TwSQS-F and TwSQS-R. The ORF cDNA of *TwSQS* was 1242 bp, which encodes a polypeptide of 413 amino acids. The result of homologous domains search showed that TwSQS belonged to the superfamily of isoprenoid biosynthesis class 1 enzymes and had specific hit with trans-isoprenyl diphosphate synthase. The sequence comparison of TwSQS showed high identity to SQSs from other plants, including *Glycyrrhiza glabra* (BAA13083.1, 88%), *P. notoginseng* (ABA29019.1, 86%), *Glycyrrhiza uralensis* (ADG36719.1, 85%), *Diospyros kaki* (ACN69082.1), *P. ginseng* (ACV88718.1, 82%), *Centella asiatica* (AAV58897.1, 82%), *Artemisia annua* (AAR20329.1, 81%), respectively. The SQS from fungi *Candida glabrata* (BAB12207.1, 41%) was added into multiple sequence alignments as an outgroup. Multiple sequence alignments indicated that TwSQS had six homologous signature regions (I–VI, Figure 1) which were considered to be the binding, catalytic and regulatory sites of SQSs. Region II and IV contained two aspartate-rich motifs (DXXXD) which were deemed to coordinate and facilitate FPP binding by binding Mg^2+^ [31,32]. Even though region VI showed low sequence similarity among the SQSs in alignment, they all had highly hydrophobic amino acid residues and performed the similar functions of membrane targeting and anchoring [32]. The TMHMM result showed that TwSQS has two transmembrane helices domains, containing residues 281–303 aa and 387–404 aa, respectively. A neighbour-joining phylogenetic tree was established to investigate the relations based on the sequences of TwSQS and other SQSs from different organisms (Figure 2). The phylogenetic tree was divided into six main clusters: eudicots, monocots, gymnosperm, algae, animal, and fungi, and TwSQS clustered within the eudicots corresponding to taxonomic classification. The result showed that TwSQS was closely related to those from *Glycyrrhiza glabra* and *Glycyrrhiza uralensis*. 

### 2.2. Purification and Identification of Recombinant Protein

To understand the catalytic function of TwSQS, it was essential to yield a certain amount of soluble protein. It was reported that removal of the C-terminal hydrophobic region could enhance the soluble expression of recombinant SQS [21]. Therefore, the truncated TwSQS without the last 28 amino acids of the C-terminal was inserted into vector pET-30a. The recombinant expression plasmid pET-30a-TwSQS was extracted and transformed into *E. coli Transetta* (DE3) to produce recombinant protein containing a His-tag at the N-terminus. The pET-30a empty vector was transformed into *E. coli Transetta* (DE3) and served as a control. After induced expression, extraction and purification, the crude protein and purified recombinant protein were analyzed by SDS-PAGE and Western blotting.

**Figure 3:**
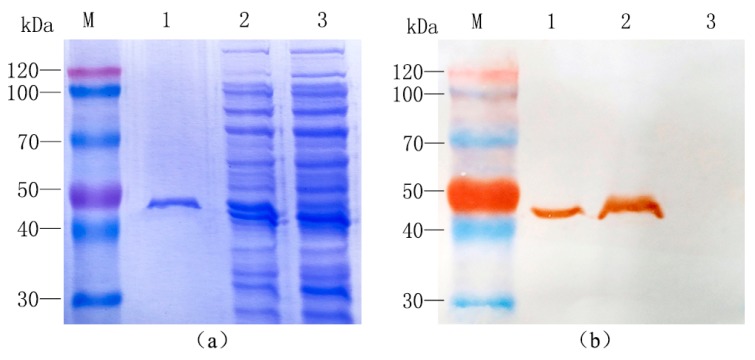
Identification of recombinant TwSQS protein. (**a**) SDS-PAGE detection of recombinant TwSQS protein expressed in *E. coli Transetta* (DE3); (**b**) Western Blot assay of the recombinant TwSQS protein expressed in *E. coli Transetta* (DE3). M: protein marker. Lane 1: purified protein of induced pET30a-TwSQS bacteria; Lane 2: supernatant of induced pET30a-TwSQS bacteria; Lane 3: supernatant of induced pET30a bacteria.

SDS-PAGE analysis showed that TwSQS was successfully expressed in the supernatant of lysates, with a molecular weight of around 49.41 kDa (Figure 3a). The corresponding protein band was also found at the same position (about 49.41 kDa) in the purified protein. Western blot detection showed that the TwSQS could specifically combine with anti-His-tag antibodies (Figure 3b). Two single immunoreactive bands were detected from the crude and purified protein of pET-30a-TwSQS bacteria. No such band was found in the crude protein of the control strain. These results indicated that the recombinant protein was successfully expressed and purified. 

### 2.3. Functional Characterization of the TwSQS

**Figure 4:**
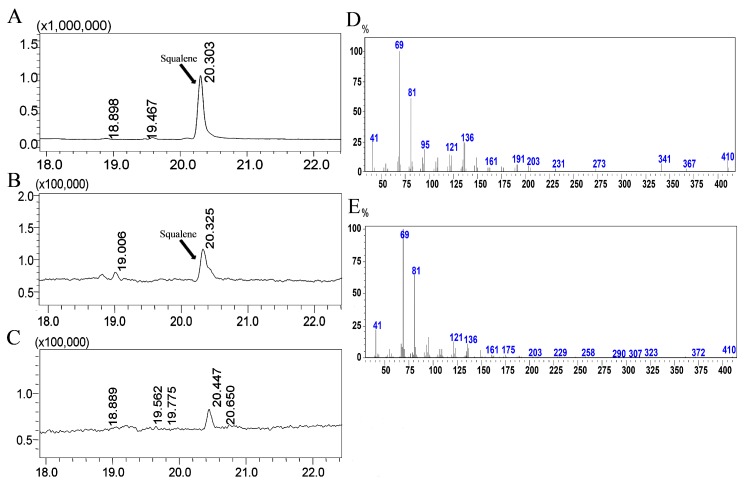
GC–MS analysis of squalene in the catalytic products of TwSQS. (**A**) GC detection of the standard squalene; (**B**) GC detection of the reaction products of TwSQS; (**C**) Control (crude protein of the control strain); (**D**) The mass spectrum of standard squalene; (**E**) MS analysis of the catalytic products of TwSQS.

To verify the function of TwSQS, the conversion activity from FPP to squalene was assayed using the purified protein with the cofactor NADPH and Mg^2+^. The crude protein of the control strain was used as a control. The catalytic products were analyzed by GC-MS (Figure 4). The peak at 20.3 min in the GC profile of TwSQS reaction products (Figure 4B) corresponded to that observed in a standard squalene sample (Figure 4A), but no such peak was detected in the control (Figure 4C). Moreover, the mass spectrum characteristic peaks (*m*/*z* = 69, 81) of the 20.3-min peak of TwSQS (Figure 4E) were consistent with those of standard squalene (Figure 4D), while no corresponding mass fragments was observed in the control. These results illustrate that *TwSQS* encodes a functional squalene synthase, converting two molecules of FPP to squalene.

### 2.4. Expression Analysis of TwSQS

**Figure 5:**
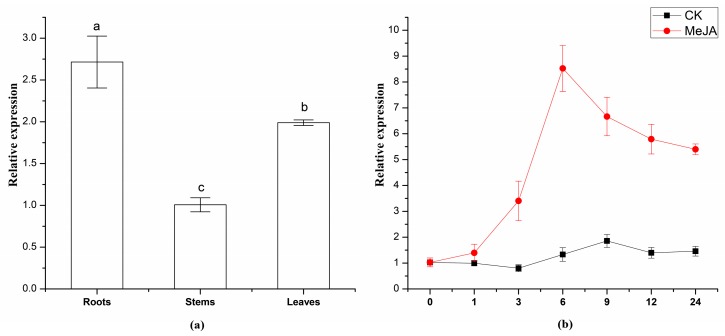
Expression analysis of *TwSQS*. (**a**) Expression analysis of *TwSQS* in different tissues of *T. wilfordii*; (**b**) Expression levels of *TwSQS* in hairy roots after MeJA treatment. Data are means ± SEM (*n* = 3). Letters on plots indicate significant difference according to Duncan’s multiple range test at *p* < 0.05. Me JA: methyl jasmonate-treated group; CK: untreated control group.

In order to study the expression pattern of *TwSQS*, we measured the *TwSQS* expression level in different tissues of *T. wilfordii* including roots, stems and leaves. The transcript levels of *TwSQS* were observed in these three tissues of *T. wilfordii* (Figure 5a). The expression level of *TwSQS* in stems was regarded as a control while the *TwSQS* expression in leaves and roots was evaluated compared with the stems. There were significant differences among the expression levels of the three tissues using Duncan’s multiple range test at *p* < 0.05 in SPSS (IBM, Armonk, NY, USA). The expression level of *TwSQS* was significantly higher than that of stems and leaves (Figure 5a).

In our previous studies, we found that 100 μM MeJA treatment could significantly increase the content of secondary metabolites and the expression level of related genes in *Tripterygium wilfordii* [12,33]. In order to determine the expression profile of *TwSQS* under MeJA, qRT-PCR analysis was performed. The result showed the *TwSQS* expression levels were significantly enhanced by 100 μM MeJA treatment in the hairy root cultures (Figure 5b). The *TwSQS* expression level before induction was regarded as a control and the *TwSQS* expression in other times of induction were calculated compared with the 0 h. After induced by MeJA, the levels of *TwSQS* expression raised rapidly at first, peaked at 6 h, and then gradually decreased. During the same period, there were no significant differences in the untreated control groups. The result indicated that MeJA can induce the expression of *TwSQS*. 

## 3. Discussion

Celastrol is a representative molecule of quinone-methide triterpenoids which are deemed as the chemotaxonomic markers of Celastraceae [3]. Because of its important medicinal activity, many studies have focused on the identification of some specific enzymes involved in celastrol biosynthesis from the MVA pathway. Squalene synthase catalyzes two molecules of farnesyl diphosphate to form squalene, a key precursor of triterpenoid biosynthesis. Given its importance in terpenoid biosynthesis, SQSs were studied as key enzymes in sterol and triterpenoid biosynthesis. However, few literature studies have described the role of TwSQS in the biosynthetic pathway of celastrol in *T. wilfordii*. In the present study, we attempted to dissect the molecular biology of the celastrol biosynthetic pathway in *T. wilfordii* by cloning and characterizing *TwSQS*. In the present study, we cloned a squalene synthase gene that was involved in celastrol biosynthesis from *T. wilfordii*, which also was the first reported SQS of Celastraceae. Sequence alignment analysis of TwSQS with other plant SQS sequences showed that six highly homologous domains were present in the TwSQS, which were very important for the catalytic/functional activity of SQS [34,35]. SQS genes from *Arabidopsis thaliana*, tobacco, human and yeast all have a single predicted transmembrane helix found in the C-terminal hydrophobic sequences identified by the TMHMM program. Interestingly, TwSQS not only have a transmembrane helix in domain VI, but also have an additional transmembrane helix near domain V. This second transmembrane helix was also reported in SQS from *G. glabra*, *G. uralensis*, *P. ginseng* and *P. notoginseng*, similar to *T. wilfordii*. The neighbor-joining phylogenetic tree also showed that TwSQS was very similar to those from *G. glabra* and *G. uralensis*.

Domain VI has highly hydrophobic amino acid residues, which is consistent with a membrane anchoring function. It were reported that deletion of the C-terminal membrane-anchoring could enhance the soluble expression of recombinant SQS [21]. This also was confirmed in our study. The soluble protein of TwSQS containing the putative full-length cDNA also was not obtained in this study (data not shown). In contrast, the soluble protein was observed in the supernatant of *E. coli* by removing the 28 amino acids of the C-terminal. In vitro enzyme reactions demonstrated that TwSQS could catalyze the reaction of FPP form to squalene under the coordination of Mg^2+^ and NADPH. Somewhat similar results were also reported in *M. officinalis* SQS [21], *S. miltiorrhiza* SQS2 [22], *P. notoginseng* SQS [24], tobacco SQS [31], and *W. somnifera* SQS [14]. In addition, the recombinant AtSQS2 which was deemed to be a non-functional SQS, was unable to synthesize squalene from FPP in the presence of NADPH and either Mg^2+^ or Mn^2+^ [18]. These reports indicated that *TwSQS* encodes a functional squalene synthase. Results of qRT-PCR analyses of *TwSQS* expression demonstrated that *TwSQS* has tissue-specific expression with highest expression in roots followed by leaves and stems (Figure 5a). Some studies found that the tissue expression pattern of SQS varies greatly in different plants. Similar patterns of gene expression were found in *S. miltiorrhiza* [22], *Euphorbia pekinensis* [36], *Taxus cuspidata* [32], while some different expression patterns were found in *B. platyphylla* [37], *W. somnifera* [38], and *Euphorbia tirucalli* [39]. In addition, several studies have reported that the MeJA could upregulates the expression of SQS [21,36]. Therefore, we investigated the expression of *TwSQS* under MeJA treatment. The expression level of *TwSQS* rapidly increased after MeJA treatment and peaked at 6 h with an eight-fold increase compared with that of the control group (Figure 5b). It is reported that celastrol mainly accumulates in the root of *T. wilfordii* and MeJA treatment could significantly increase the content of celastrol [40]. Our results, thus, provide an additional evidence that *TwSQS* is positively involved in celastrol biosynthesis. Further work is to regulate the isoprenoid biosynthesis through the overexpression and MeJA induction of the *TwSQS* gene so as to enhance the production of celastrol in *T. wilfordii.*

## 4. Materials and Methods

### 4.1. Plant Material

The different fresh tissues of *T. wilfordii* were collected from the plants grown in the greenhouse of Northwest A & F University, China. The hairy root cultures were established as described previously [41]. The hairy root of *T. wilfordii* were cultured in 250 mL Erlenmeyer flasks containing 100 mL of Murashige-Skoog liquid medium, 30 g/L sucrose. Each flask was inoculated with 1 g fresh weight of roots and maintained in darkness at 26 °C. The hairy roots were elicited with 100 μM MeJA after 25 days of cultivation. The roots were harvested at 0, 1, 3, 6, 9, 12, 24 h after MeJA treatment. All the materials were rapidly frozen using liquid nitrogen and then kept at −80 °C for RNA isolation and chemical detection. 

### 4.2. Total RNA Extraction and cDNA Synthesis

Total RNA was extracted from 100 mg of *T. wilfordii* roots using the plant total RNA extraction kit (TIANGEN Biotech Co., Ltd., Beijing, China). The RNA quality and concentration were measured by gel electrophoresis (1% agarose) and spectrophotometer analysis. The total RNA was reverse transcribed into cDNA template using Takara PrimeScript™ 1st strand cDNA synthesis kit (TaKaRa Biotechnology, Dalian, China).

### 4.3. Cloning and Bioinformatics Analyses of TwSQS

The ORF cDNA of *TwSQS* was cloned using the specific primers TwSQS-F and TwSQS-R (Table 1) which was designed according to the sequence from reported squalene synthase of *T. wilfordii* (KR401220.1). The PCR products of *TwSQS* were detected, purified and cloned into the pClone007 Blunt Simple Vector (Tsingke Biotech Co., Beijing, China) followed by sequencing.

The sequences similarities search of deduced amino acid sequence was performed through NCBI BLAST program (https://blast.ncbi.nlm.nih.gov/Blast.cgi?PROGRAM=blastp&PAGE_TYPE=BlastSearch&BLAST_SPEC=MicrobialGenomes&LINK_LOC=blasttab&LAST_PAGE=blastn). Sequence conserved domains were detected using the tool of Conserved Domains Search (http://www.ncbi.nlm.nih.gov/Structure/cdd/wrpsb.cgi). Protein Transmembrane Helical domains were analysed by TRMHMM software programs (http://www.cbs.dtu.dk/services/TMHMM-2.0/). Sequence multiple alignment was performed using the software of DNAMAN 8.0 (Lynnon Biosoft, Quebec, QC, Canada). Phylogenetic analysis was established using the Neighbor-Joining method by MEGA 6.06 (Biodesign Institute, Tempe, AZ, USA). The confidence values of the branches were evaluated by bootstrap analysis with 1000 replicates.

### 4.4. Expression of Recombinant Protein

Squalene synthase is a membrane bound enzyme that has two transmembrane helices at the carboxy-terminal region. The second transmembrane helices is not located in the conserved domains. The C-terminal has highly hydrophobic amino acid residues, which is consistent with a membrane anchoring function. It was reported that removal of the C-terminal hydrophobic region could enhance the soluble expression of recombinant SQS [31]. It may be because removal of the C-terminal hydrophobic region could enhance the water-solubility of recombinant SQS and reduce the ability of SQS to be targeted to the membrane. So the carboxy-terminal truncated *TwSQS* was amplified using the designed site primers TwSQS-TF (*BamH*I) and TwSQS-TR (*Sac*I) (Table 1).

The PCR product and the expression vector pET-30a were separately double-digested with *BamH*I and *Sac*I, and ligated by T4 ligase. The recombinant vector, pET-30a-TwSQS, was verified by sequencing. pET-30a-TwSQS was transformed into competent *E. coli Transetta* (DE3) to produce recombinant protein that containing a His-tag at N-terminus. The transformed bacteria was grown to A600 = 0.6 at 37 °C in LB medium. Expression of TwSQS was induced with 0.8 mM Isopropyl β-d-1-thiogalactopyranoside (IPTG) at 28 °C. After culturing for 6 h, the cells were collected by centrifuging at 6000× *g* for 10 min, and then washed 3 times with 0.05 M Tris–HCl (pH 7.5). The cells were resuspended in extraction buffer (50 mM Tris–HCl, pH 7.5, 10% glycerol, and 5 mM DTT) and lysed by sonication. The crude enzyme solution was harvested after centrifugation at 12,000× *g* at 4 °C for 30 min.

### 4.5. Purification of Expressed TwSQS and Western Blot Analysis

The crude enzyme solution was subjected to His-Tag purification using a Ni-NTA agarose gravity column (TransGen Biotech, Beijing, China) [42]. Ni-NTA agarose was equilibrated with balance buffer (10 mM imidazole, 50 mM Tris–HCl, pH 7.5, 50 mM NaH_2_PO_4_, and 0.3 M NaCl). The crude enzyme solution was added to the column and then washed with balance buffer. 

The desired protein was eluted from Ni-NTA resin with increasing imidazole concentration (50–200 mM) in elution buffer. The eluted fractions were analyzed on a 10% SDS-PAGE gel and stained by Coomassie Blue R250 solution. The imidazole of purified protein was removed by centrifugal ultrafiltration (Amicon Ultra 10 kDa, Millipore, Burlington, MA, USA).

The recombinant protein was verified via Western blot analysis. Protein bands were transferred onto nitrocellulose membrane from SDS-PAGE gel. The membrane was blocked with 5% (*w*/*v*) skimmed milk in TBST (triethanolamine buffered saline adding 0.05% Tween 20) for 2 h, and then incubated with HRP Conjugated Anti His-Tag Mouse Monoclonal Antibody (1:3000, CWBIO, Beijing, China) at 4 °C overnight. After washing five times with TBST (5 min each time), the blots were revealed with a diaminobenzidine (DAB) kit (Solarbio, Beijing, China).

### 4.6. In Vitro Enzyme Reaction

The enzyme reaction of the recombinant protein TwSQS was conducted as follows. The purified protein incubated at 30 °C with 50 μM FPP triammonium salt (Sigma–Aldrich, St. Louis, MO, USA), 50 mM Tris–HCl (pH 7.5), 25 mM MgCl_2_, 1 mM DTT, 2% Glycine and 3 mM NADPH in a total reaction volume of 300 µL. After 8 h, the reaction product was extracted four times with 400 µL *n*-hexane and then analyzed by GC-MS. The GC-MS detection was performed on GCMS-QP2010 Ultra (Shimadzu, Kyoto, Japan) using the method described as Rong et al. [22].

### 4.7. Expression Analysis

Total RNA from different tissues and hairy roots treated by MeJA was extracted separately using the plant total RNA extraction kit (TIANGEN Biotech Co., Ltd., Beijing, China), and reversely transcribed into cDNA using the Prime Script™ RT reagent kit with gDNA Eraser (TaKaRa). The qRT-PCR analysis was performed using the SYBR Premix Ex Taq (TaKaRa) on a CFX96™ Real-time PCR system (Bio-Rad, Hercules, CA, USA). Elongation factor 1 α (EF1α) was used as a reference gene for gene expression normalization [43]. Primers used for expression analysis of *TwSQS* (TwSQS-qF and TwSQS-qR) and EF1α (Efα-F and Efα-R) were given in Table 1. Each analysis included three independent experiments and two biological replicates. The relative amounts of *TwSQS* were quantified by using 2^−ΔΔ*C*t^ method [44].

## 5. Conclusions

We have successfully cloned and characterized a squalene synthase gene, which is involved in celastrol biosynthesis in the medicinal plant *T. wilfordii*. The recombinant TwSQS protein was expressed in *E. coli* and purified. After determination of squalene by GC-MS, we verified that the *TwSQS* encodes a functional squalene synthase. Furthermore, the expression profile of the *SQS* gene in different tissues and methyl jasmonate treatments were determined. The results indicate that *TwSQS* is positively involved in celastrol biosynthesis. The present study is helpful to understand celastrol in *T. wilfordii* at the molecular level. Further studies on TwSQS would be useful for understanding celastrol biosynthesis and would provide molecular resources for the biotechnological improvement of this important medicinal plant.

## Figures and Tables

**Table 1:** Primers used in this study.

Primer Name	Primer Sequence 5′→3′
SQS-F	ATGGGGAGTTTGTGGACGA
SQS-R	ACTCGGGGGCTGACATCC
SQS-TF	CGGGATCCATGGGGAGTTTGTGGACGA
SQS-TR	CGAGCTCATGACTCGCCATTAACTATGCC
SQS-QF	GCGCTGCGAGATCCAGCCAT
SQS-QR	TGCAGTGAGACCACGCCTCAT
EF1α-F	CCAAGGGTGAAAGCAAGGAGAGC
EF1α-R	CACTGGTGGTTTTGAGGCTGGTATCT
